# Transactions Annual Meeting Illinois State Dental Society

**Published:** 1884-09

**Authors:** 


The transactions of the 20th Annual Meeting of the Illinois State Dental Society has just come to hand. It is a well gotten up volume of 170 pages, containing in full the proceedings of the meeting held at Springfield May 13th. It contains the proceedings, papers, and discussions. Presents matter of great interest to the profession. The papers are fully abreast with the times, containing fresh and interesting matter, and afford material for thought and study for the practitioner. The volume is neatly gotten up in every respect, bound in cloth. It can be obtained by applying to the Secretary, Dr. J. W. Wassall, of Chicago.

